# Effect of New Zealand Blackcurrant Extract on Isometric Contraction-Induced Fatigue and Recovery: Potential Muscle-Fiber Specific Effects

**DOI:** 10.3390/sports8100135

**Published:** 2020-10-15

**Authors:** Mark E. T. Willems, Megan Bradley, Sam D. Blacker, Ian C. Perkins

**Affiliations:** Institute of Sport, University of Chichester, College Lane, Chichester PO19 6PE, UK; meganbradley263@gmail.com (M.B.); s.blacker@chi.ac.uk (S.D.B.); i.perkins@chi.ac.uk (I.C.P.)

**Keywords:** blackcurrant, anthocyanins, muscle force, muscle fatigue, twitch force, recovery, sports nutrition

## Abstract

New Zealand blackcurrant (NZBC) extract has shown performance-enhancing effects during cycling, running and sport climbing. We examined effects of NZBC extract on (1) voluntary and twitch force of the *quadriceps femoris muscles* during repeated isometric contraction-induced fatigue, (2) twitch force during recovery and (3) muscle fiber-specific effects. Familiarized recreationally active males (n = 12, age: 24 ± 5 yrs; height: 180 ± 5 cm; body mass: 89 ± 11 kg) performed sixteen, 5-s voluntary maximal isometric contractions (iMVC) separated by 3-s rest. Twitch force was recorded before, during the 3-s rests and 5-min recovery. Supplementation consisted of 7-days intake of NZBC extract (600 mg∙day^−1^ containing 210 mg anthocyanin) in a double-blind, randomized, placebo-controlled crossover design with a 14-days washout. NZBC extract allowed for greater force in the first quartile of the iMVCs. Twitch force at baseline was 12% higher with NZBC extract (*p* = 0.05). However, there was no effect of NZBC for twitch force during the 16-iMVCs and recovery. Based on the maximum post-activation potentiation during the placebo 16-iMVCs, four subjects were classified of having a predominant type I or II muscle fiber typology. In type II, NZBC extract provided a trend for increased MVC force (~14%) in the first quartile and for type I in the fourth quartile (~10%). In type I, NZBC extract seemed to have higher twitch forces during the fatiguing exercise protocol and recovery, indicating increased fatigue resistance. New Zealand blackcurrant extract affects force during repeated maximal isometric contractions. Future work on mechanisms by NZBC extract for muscle fiber-specific fatigue-induced force responses is warranted.

## 1. Introduction

In recent years, studies have examined the ergogenic potential of fruit and vegetable derived components. For example, 6 days of nitrate-rich beetroot juice provided performance-enhancing effects for severe-intensity cycling exercise to exhaustion [1]. For fruit-derived supplements (e.g., blackcurrant, blueberry, Montmorency cherry, pomegranate), the efficacy for performance-enhancement is associated with the polyphenol composition [2]. Blackcurrant is a berry with a high content of primarily four anthocyanins. In general, an anthocyanin is a flavonoid with potential anti-oxidant and anti-inflammatory properties providing health benefits [3]. Studies with anthocyanin-rich New Zealand blackcurrant extract have provided observations on the performance-enhancing effects for a 16.1 km cycling time trial [4], repeated high-intensity treadmill running [5], two 4 km cycling time trials [6], repeated sprints in the running-based anaerobic sprint test [7], maximal sprints in the Loughborough Intermittent Shuttle Test [8], and sport climbing [9]. The mechanisms for the performance-enhancing effects of New Zealand blackcurrant extract are unknown, but may be due to enhanced blood flow [10] and muscle oxygenation [11]. However, cognitive effects by intake of blackcurrant cannot be excluded [12]. All the studies on the performance-enhancing effects of New Zealand blackcurrant extract had a 7-day dosing strategy with either 105 or 210 mg of anthocyanins per day. Therefore, it is possible that the build-up and presence of anthocyanins and anthocyanin-derived metabolites affect physiological fatigue mechanisms and cell function. Post-exercise recovery, especially when the exercise is of maximal or high-intensity, can be characterized by regaining muscle performance from exercise-induced fatigue [13], and many studies have examined the effects of polyphenol intake on post-exercise recovery. For example, in the days following prolonged intermittent sprint activity, Montmorency cherry juice accelerated functional recovery [14], but precise recovery mechanisms are also unknown.

Mechanisms for exercise-induced fatigue are primarily dependent on the exercise duration and exercise intensity and can have central (i.e., located in the central nervous system) and peripheral (i.e., located in the muscle) components [15]. In addition, exercise-induced fatigue by intermittent maximal-intensity exercise is affected by the muscle fiber composition of the contracting skeletal muscles [16,17]. In general, slow-twitch (type I) muscle fibers are more fatigue-resistant than the fast-twitch (type II) muscle fibers. Hamada et al. [13] observed that males with a predominant fast-twitch muscle fiber-type profile in the *vastus lateralis muscle* had greater decreases during repeated maximal voluntary isometric contractions (i.e., type II: 49.6% vs. type I: 22.8%, respectively). The study by Lievens et al. [17] confirmed that the decrease in cycling power by three 30-s Wingate tests was also larger in males with a predominant type II profile (i.e., type II: 61%% vs. type I: 41%, respectively). In addition, muscle fiber typology also affected the recovery from the three 30-s Wingate tests [17].

As evidence is building that fruit- and vegetable-derived components have the potential to enhance performance by postponing or limiting the effects of exercise-induced fatigue, it would be of interest to examine whether the effect of a nutritional ergogenic aid is fiber type specific. For nitrate-rich beetroot juice, for example, the effect is predominantly in the severe-intensity exercise domain and suggests specific effects on metabolic control in type II muscle fibers [18]. It can be theorized, then, that individuals with a predominant type II fiber typology would respond differently than those with a predominant type II fiber typology. Such information may inform personalized nutritional strategies. The studies that reported the performance-enhancing effects of the intake of New Zealand blackcurrant extract involved whole-body exercise modalities (e.g., cycling [4] and treadmill running [5]) and did not consider muscle fiber typology. Optimal performance of maximal or high-intensity exercise always requires high motivation and the observed decline of muscle force by exercise-induced fatigue may be partly due to non-peripheral (i.e., not in the muscle) physiological factors. However, muscle twitches evoked by surface electrical stimulation of skeletal muscles allow for the quantification of contraction- and fatigue-induced responses with fiber-type specific effects [13]. The effects of an anthocyanin-rich supplement on the performance of an isolated muscle group during exercise-induced fatigue and recovery are not known. In addition, it is not known whether the response of individuals with a predominant type I or type II muscle fiber type profile is differentially affected by the intake of an anthocyanin-rich supplement. Differences in capillarization between muscle fiber types, and the potential of blackcurrant intake enhancing blood flow, may suggest that a fiber-type specific response is possible.

Therefore, the primary aim of the present study was to examine the effect of anthocyanin-rich New Zealand blackcurrant extract on (1) voluntary force production during a series of maximal isometric maximal contractions, (2) twitch force during the fatiguing isometric exercise protocol, and (3) twitch force during recovery from a fatiguing isometric exercise protocol. The secondary aim was to analyze the fiber-type specific responses of voluntary isometric force and twitch force for individuals categorized by a predominant fiber type based on the observations of Hamada et al. [13].

## 2. Materials and Methods

### 2.1. Participants and Experimental Design

Healthy recreationally active males (n = 12, age: 24 ± 5 yrs; body mass: 89 ± 11 kg, height: 180 ± 5 cm) volunteered for the study. Participants were informed about the aim and the experimental procedures of the study before providing written informed consent. Participants were not taking any additional supplements during the study, had no history of knee and ankle joint problems, and no known allergy to berries. Ethical approval was obtained from the University of Chichester Ethics Committee with all procedures in accordance with the Declaration of Helsinki by the World Medical Association (ethical approval code No. 1617_05). The study had a randomized, placebo-controlled, double-blind, cross-over design. The main goal of the experiments was to determine whether there was an effect of New Zealand blackcurrant extract on the force of the *quadriceps femoris muscles* during maximal voluntary contractions and surface electrical stimulation-induced twitch force by performing a fatiguing exercise protocol. The fatiguing exercise protocol was similar to the non-invasive protocol in the study by Hamada et al. [13], and would allow the identification of individuals with a type I or type II predominant muscle fiber typology [19]. In brief, Hamada et al. [19] used needle biopsies of the *vastus lateralis muscle* and showed that fiber type composition was associated with the amount of post-activation potentiation using a 10 s maximal voluntary contraction. Subsequently, Hamada et al. [13] observed the muscle fiber-type related difference in twitch and iMVC profiles during a fatiguing exercise protocol. Participants were familiarized for force measurements of the *quadriceps femoris muscles* and the fatiguing exercise protocol and surface electrical stimulation.

### 2.2. Mechanical Testing and Surface Electrical Stimulation of the Quadriceps Femoris Muscles

Participants visited the laboratory for three morning sessions at the same time of day. Participants sat on a custom-build chair with the upper body firmly against the back of the chair by use of two Velcro straps over the chest and belly. During force measurements, the participants were still instructed not to lean forward with the upper body and keep arms crossed across their chest. A polyester strap with a custom-made metal clamp was positioned around the lower right leg above the medial malleolus. A steel chain was attached to the metal clamp and allowed the ankle to be connected with a calibrated s-beam load cell (RS 250 kg, Tedea Huntleigh, Cardiff, UK). The force of the *quadriceps femoris muscles* was measured with a sampling frequency of 1000 Hz using a PowerLab data acquisition system (ADinstruments Ltd., Oxford, UK) and displayed using Chart for Windows (Chart 4 V4.1.2, AD Instruments, Oxford, UK) on a computer screen positioned about 1.5 m in front of the participants.

In the familiarization session, the current for maximal twitch force was established. Twitch force was measured using surface electrical stimulation with two custom made electrodes (13 cm × 20 cm) positioned on the proximal and distal part of the upper leg. The electrodes were connected to a constant current stimulator (Model DS7AH, Digitimer Limited, Welwyn Garden City, UK) and controlled by a Neurolog pulse generator (Model NL900D, Digitimer Limited, Welwyn Garden City, UK). Subsequently, participants practiced the execution of maximal isometric voluntary contractions and completed the fatiguing exercise protocol. In brief, the fatiguing exercise protocol by Hamada et al. [13] consists of 16-iMVCs of 5 s each with a 3 s rest period. Twitch force was measured before the iMVCs and 2 s into the rest period between iMVCs. After completion of the 16-iMVCs, a 5 min recovery period commenced with only measurement of twitch force at 5 s and 30 s and then subsequently at 30 s intervals until the completion of the 5 min recovery [13]. Post-activation potentiation by the fatiguing exercise protocol was quantified as the % change of the highest twitch force during the protocol compared to the baseline twitch force (i.e., recorded before the start of the fatigue exercise protocol). In experimental visits two and three, participants performed the fatiguing exercise protocol of 16-iMVCs and recovery with twitch force recordings.

### 2.3. New Zealand Blackcurrant Extract Supplementation

Seven days before the experimental visits two and three, participants were required to consume 600 mg (i.e., 2 capsules of 300 mg) of New Zealand blackcurrant extract. For the first six days, participants were instructed to take one capsule in the morning and one capsule in the evening. Each capsule contains 105 mg of blackcurrant anthocyanins (i.e., delphinidin-3-*O*-rutinoside, 35–50%; delphinidin-3-*O*-glucoside, 5–20%, cyanidin-3-*O*-rutinoside, 30–45% and cyanidin-3-*O*-glucoside, 3–10%) with the remaining content natural plant sugars (CurraNZ^TM^, Health Currancy Ltd., Surrey, UK; CurraNZ Ltd., Auckland, New Zealand). The dosing strategy was based on dose–response observations of New Zealand blackcurrant extract effects by Cook et al. [20]. The dosing strategy had no side effects. For the placebo condition (PLA), participants were taking visually-matched placebo capsules (2 × 300 mg microcrystalline cellulose M102). The final dose of two capsules was taken two hours before arriving in the laboratory and two hours after a light breakfast (one slice of bread and water). There was a 14 day wash out period between experimental sessions two and three. We did not record the participant’s perception whether they had taken placebo or NZBC extract.

### 2.4. Physical Activity and Dietary Analysis

Nutritional intake for participants was not controlled. However, participants were instructed to replicate their two-day dietary intake before experimental session two and three, but dietary intake was not quantified in the present study. Participants completed a food frequency questionnaire on their normal daily intake of fruit, vegetables and drinks to quantify habitual anthocyanin intake using Phenol Explorer [21], a database on polyphenol content in foods (http://phenol-explorer.eu). Daily anthocyanin intake was calculated by the sum of consumption frequency of each food multiplied by the anthocyanin for the portion size estimate and estimated to be 51 ± 44 mg·day^−1^. For preparation of the testing sessions, participants were instructed to refrain from strenuous exercise for 48 h prior, no alcohol intake for 24 h prior, and no caffeine on the day of testing. All testing was in the morning and only the light breakfast and the final two capsules was allowed prior to arrival at the laboratory.

### 2.5. Statistical Analysis

Graphpad Prism (version 5.00 for Windows, GraphPad Software, San Diego, CA, USA) was used for statistical analyses. Normal distribution was checked with the D’Agostino and Pearson omnibus test. A two-way ANOVA was performed on the forces during the fatiguing exercise protocol and recovery to analyses interaction and main effects of supplementation on maximum voluntary force of the 16-iMVCs protocol and force of the 27 twitches for the group data and data for participants that were selected to have a predominant type I or II muscle fiber type profile. A paired samples *t*-test was used for (1) the force of the first twitch, i.e., before the fatiguing exercise protocol, (2) the iMVC quartile data, (3) the force of the twitches during the fatiguing exercise protocol compared to the force of the first twitch, and (4) average twitch force during recovery. Quartile analysis is common in studies with sustained isometric contractions to exhaustion (e.g., [22]) and allowing observations for time periods with different fatigue development [23]. Statistical significance was set at *p* < 0.05. Data are reported as mean ± SD and 95% confidence intervals (95% CI). Interpretation of 0.05 > *p* ≤ 0.1 is considered to be a trend [24]. For significant and trend changes, Cohen’s *d* was calculated and considered trivial (*d* < 0.2), small (*d* = 0.2–0.39), moderate (*d* = 0.4–0.69) and large (*d* ≥ 0.7), respectively.

## 3. Results

### 3.1. Performance of Maximal Voluntary Isometric Contractions during the Fatiguing Exercise Protocol

**Figure 1:**
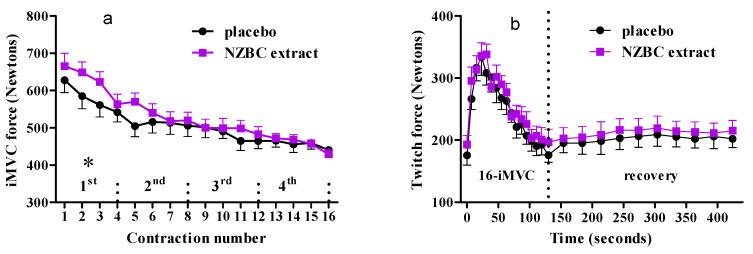
(**a**) Voluntary maximum isometric force during the repeated 16 voluntary maximal isometric contractions (iMVCs). (**b**) Twitch forces during the repeated 16-iMVCs and recovery. For clarity, force data are presented as mean ± SEM. 1st, 2nd, 3rd and 4th represent the quartiles. * indicates a difference between conditions in the 1st quartile *p* = 0.047).

An analysis of the repeated iMVCs showed no interaction and condition effect, but a change with contraction number. The decrease in maximal voluntary isometric force of *quadriceps femoris muscles* with contraction number is expected (Figure 1a) as participants performed the fatiguing exercise protocol by Hamada et al. [13]. However, analysis of quartiles showed that NZBC extract allowed for higher total force production of 9 ± 12% (range: −17% to 25%) by the *quadriceps femoris muscles* in the first quartile of the repeated 16-iMVCs (PLA: 2316 ± 416 N, 95% CI [2051, 2580]; NZBC extract: 2501 ± 381 N, 95% CI [2258, 2743], *p* = 0.047) (Figure 1a), with a moderate effect size (*d* = 0.46). Eleven participants (~92%) had higher total force production with six participants having an increase by more than 10%. In addition, in the placebo condition, the second iMVC was already lower than the first iMVC (*p* = 0.018) with small effect size (*d* = −0.36), whereas in the NZBC extract condition, the third iMVC was the first contraction lower than the first iMVC *p* = 0.040), also with small effect size (*d* = −0.39). However, there were no differences for total force production in the 2nd (*p* = 0.198), 3rd (*p* = 0.294) and 4th quartile (*p* = 0.905). It is concluded that New Zealand blackcurrant extract can increase maximal isometric force production of skeletal muscles during a fatiguing exercise protocol.

### 3.2. Twitch Forces during Fatiguing Exercise Protocol and Recovery

Maximal twitch force at baseline was 12% higher with NZBC extract (*p* = 0.05), with a small effect size (*d* = 0.32), and with 9 participants (75%) having higher values. An analysis of the twitch forces showed no interaction and condition effect, but a change over time (Figure 1b). The initial increase in twitch force during the fatiguing exercise protocol (Figure 1b) is due to post-activation potentiation and the maximum post-activation potentiation was not different between placebo (118 ± 97%, 95% CI [56, 179]) and NZBC extract (102 ± 84%, 95% CI [49, 155]) (*p* = 0.181). During recovery, there was no difference for the mean twitch force (PLA: 200 ± 57 N, 95% CI [164, 236], NZBC extract: 211 ± 60 N, 95% CI [172, 249], *p* = 0.365).

### 3.3. Fiber-Type Specific Responses on Performance of Maximal Voluntary Isometric Contractions during Fatiguing Exercise Protocol

Based on the maximum post-activation potentiation during the placebo 16-iMVCs, the four participants with the lowest and highest maximum post-activation potentiation were taken as those with a type I and type II fiber predominant typology [13]. In agreement with observations by Hamada et al. [13], the changes in iMVC during the 16-iMVC fatiguing protocol for type I and II fiber predominant typology were different (*p* = 0.026), and 21 ± 8% (range 10–30%) and 34 ± 3% (range 31–37%), respectively.

**Figure 2:**
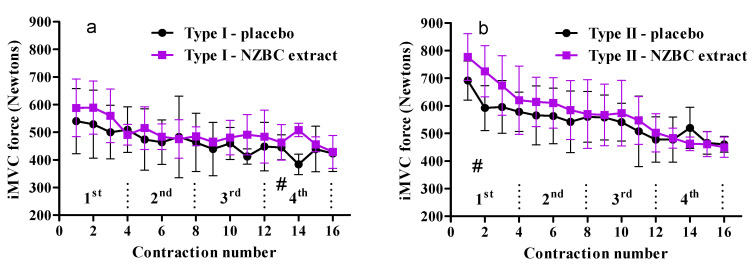
Voluntary maximum isometric force during the repeated 16-iMVCs for type I (**a**) and type II (**b**) fiber predominant typology. # indicates a trend for a difference between conditions in the 1st quartile (*p* = 0.064) and 4th quartile (*p* = 0.094).

In the first quartile (Figure 2), total force production was not changed for type I (PLA: 2079 ± 407 N, NZBC extract: 2229 ± 319, *p* = 0.155), but there was a trend to be higher by 14% for type II (PLA: 2458 ± 297 N, NZBC extract: 2796 ± 399 N, *p* = 0.064), with a large effect size (*d* = 0.96). In the second quartile, total force production was not changed for type I (PLA: 1885 ± 433 N, NZBC extract: 1960 ± 217 N, *p* = 0.629) and type II (PLA: 2231 ± 405 N, NZBC extract: 2380 ± 405 N, *p* = 0.461). In the third quartile, total force production was not changed for type I (PLA: 1759 ± 259 N, NZBC extract: 1921 ± 226 N, *p* = 0.101), and for type II (PLA: 2084 ± 339 N, NZBC extract: 2190 ± 379 N, *p* = 0.105). In the fourth quartile, there was a trend for total force production to be higher by 10% for type I (PLA: 1692 ± 211 N, NZBC extract: 1857 ± 133 N, *p* = 0.094), with a large effect size (*d* = 0.94) and no change for type II (1924 ± 218 N, 1856 ± 114 N, *p* = 0.336) (Figure 2). These observations suggest that New Zealand blackcurrant extract allows higher force production for type II muscle fiber predominant typology in a relatively unfatigued state but for type I muscle fiber typology when relatively fatigued. It may also suggest an increased fatigue resistance for type I fiber predominant typology caused by the intake of New Zealand blackcurrant extract.

### 3.4. Fiber-Type Specific Responses on Twitch Forces during Fatiguing Exercise Protocol and Recovery

**Figure 3:**
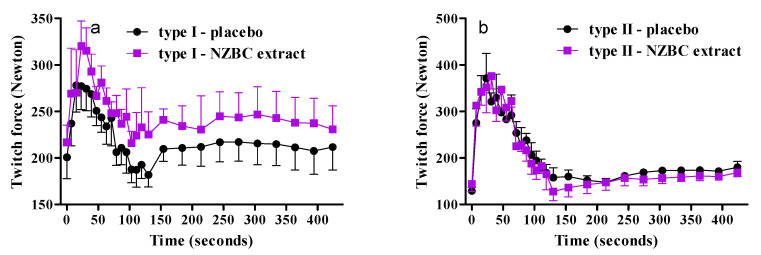
Twitch force between the repeated 16-iMVCs and during recovery for type I (**a**) and type II (**b**) muscle fiber predominant typology. For clarity, data are presented as mean ± SEM.

Figure 3 shows the absolute twitch forces for type I and type II muscle fiber predominant typology. An analysis of the twitch forces for type I and type II muscle fiber predominant typology showed no interaction and condition effect, but a change over time (Figure 3). For type I, the twitch forces with NZBC extract seem to be higher during the 16-iMVC fatiguing protocol and recovery, suggesting an increased fatigue resistance. It is possible that the selection of only four participants with a suggested type I and II muscle fiber predominant typology limits the power of the study. 

## 4. Discussion

The novel finding of the present study is the ergogenic potential of New Zealand blackcurrant extract to enhance force production of repeated maximal isometric contractions of *quadriceps femoris muscles*. In addition, there seems to be a muscle fiber-type specific effect on the force production by repeated maximal isometric contractions. For type I muscle fiber predominant typology, the benefits for enhanced force production by New Zealand blackcurrant extract appear when there is already substantial fatigue in comparison with type II muscle fiber predominant typology, for which the benefits appeared early on in the fatiguing exercise protocol when there was less fatigue. Our observations on twitch force also suggest an apparent increased fatigue resistance for type I fiber predominant typology with intake of New Zealand blackcurrant extract. Such observations, although obtained for an in-vivo isolated muscle group, may suggest that NZBC extract has the potential to enhance performance for exercise with different intensities and duration. Enhanced maximal force production may be beneficial for brief bursts of sprint activity in sport. The increased fatigue resistance may be of benefit for high-intensity long duration exercise. The observations in the placebo condition in the present study are consistent with muscle fiber-specific observations in previous studies, i.e., (1) higher iMVC forces, (2) higher post-activation potentiation, and (3) more contraction-induced fatigue in subjects with type II muscle fiber predominant typology [13]. 

In the present study, the fatiguing exercise protocol consisted of 16 repeated maximal isometric contractions of 5 s with the *quadriceps femoris muscles* [13]. Fatigue induced by repeated maximal isometric contractions has central and peripheral origins [25]. One 5 s isometric contraction of *quadriceps femoris muscles* at two-thirds of maximal intensity performed after occlusion release lowers phosphocreatine by ~25% [26]. Although the 3 s rest period between the maximal isometric contractions allows for some resynthesis of phosphocreatine, the breakdown and lowering of phosphocreatine [25] is accompanied by an accumulation of inorganic phosphate impairing force production by cross-bridges [27]. Resynthesis of phosphocreatine benefits from enhanced blood flow and muscle reoxygenation [28]. Interestingly, NZBC extract appears to improve muscle oxygenation during intermittent exercise [11] and blackcurrant can enhance blood flow [29]. Although type II muscle fibers have a higher content of phosphocreatine [30,31], the decline in phosphocreatine during maximal-intensity exercise is higher in type II muscle fibers [31]. Therefore, it is possible that the decline in phosphocreatine combined with impaired phosphocreatine resynthesis in type II muscle fibers may explain a disappearance of an ergogenic effect when the muscles became more fatigued. In addition, any vasodilatory effects by blackcurrant intake may benefit more the type I muscle fibers as they have higher local capillary to fiber ratio than type 2 muscle fibers [32]. Therefore, a potential increase in blood flow by intake of blackcurrant [10,29] may have a larger beneficial effect on type I muscle fibers. Interestingly, type I muscle fiber predominant typology shows enhanced force production later in the fatiguing exercise protocol. However, because the group observations indicate an effect of NZBC extract on iMVC forces but not twitch forces, it is possible that voluntary performance may be enhanced via a central mechanism. Many studies have reported performance-enhancing effects by intake of New Zealand blackcurrant extract [4,5,6,7,8,9]. It is possible that the variation in individual responses to New Zealand blackcurrant extract in previous studies may be partly due to fiber-type specific effects during exercise. Future work is recommended to examine the effect of New Zealand blackcurrant extract on the responses to the repeated isometric contractions in trained sprint and long-distance athletes. Sprint and long-distance athletes have distinct differences in fiber type composition [33]. Such studies may inform personalized sport-specific advice on performance-enhancing anthocyanin-rich supplementation.

The maximum twitch force of the *quadriceps femoris muscles* in the present study was 8.5% higher with intake of NZBC extract. Our observation is similar to the effects of 7-day nitrate intake on peak twitch force, for which an increase of 7% was reported for the *quadriceps femoris muscles* using femoral nerve stimulation [34]. In Haider and Folland [34], maximal voluntary force production was not affected. However, it needs to be noted that the optimal dosing strategy of New Zealand blackcurrant extract for performance enhancement is not known. The enhanced maximum twitch force may be due to improved calcium handling and/or increased cross-bridge sensitivity to calcium. In the present study, the higher maximum twitch force was observed for the group data and did not seem to be clearly present for the analysis of muscle fiber-specific effects. However, for type I muscle fiber predominant typology, twitch forces during the fatiguing exercise protocol and recovery seemed to be higher with NZBC extract. Therefore, it seems that NZBC extract can enhance the phosphorylation of myosin regulatory light chain. In effect, calcium sensitivity increased, resulting in increased force production [35]. Therefore, during the fatiguing exercise protocol, it seems that higher post-activation potentiation occurs, and this phenomenon has been proposed to enhance endurance performance [36]. In addition, it is tempting to speculate that post-activation potentiation is more affected in type I muscle fibers by the intake of New Zealand blackcurrant. Future work on the effects of anthocyanin-rich supplements on muscle fiber-specific functional effects with adequate power [37] is warranted. In the present study, the post-hoc computed achieved power for the first quartile iMVC force values for the group was 0.31 and 0.86 for participants with fiber type II predominant typology. It is also recommended that observations on the within-day and between-day variability of the twitch and iMVC forces are reported in future work. 

## 5. Conclusions

Anthocyanin-rich New Zealand blackcurrant extract can enhance force production during repeated maximal voluntary isometric contractions. In addition, our observations seem to suggest that New Zealand blackcurrant extract is able to modify muscle force with muscle fiber-type specific effects.
